# Multimodal human motion dataset of 3D anatomical landmarks and pose keypoints

**DOI:** 10.1016/j.dib.2024.110157

**Published:** 2024-02-06

**Authors:** Ana Virginia Ruescas-Nicolau, Enrique José Medina-Ripoll, Eduardo Parrilla Bernabé, Helios de Rosario Martínez

**Affiliations:** Instituto de Biomecánica - IBV, Universitat Politècnica de València, Edificio 9C. Camí de Vera s/n, 46022 Valencia, Spain

**Keywords:** Body tracking, Human motion analysis, Computer vison, Machine learning, 3D temporal scanner, Gait, Simulation

## Abstract

In this paper, we present a dataset that takes 2D and 3D human pose keypoints estimated from images and relates them to the location of 3D anatomical landmarks. The dataset contains 51,051 poses obtained from 71 persons in A-Pose while performing 7 movements (walking, running, squatting, and four types of jumping). These poses were scanned to build a collection of 3D moving textured meshes with anatomical correspondence. Each mesh in that collection was used to obtain the 3D locations of 53 anatomical landmarks, and 48 images were created using virtual cameras with different perspectives. 2D pose keypoints from those images were obtained using the MediaPipe Human Pose Landmarker, and their corresponding 3D keypoints were calculated by linear triangulation.

The dataset consists of a folder for each participant containing two Track Row Column (TRC) files and one JSON file for each movement sequence. One TRC file is used to store the 3D data of the triangulated 3D keypoints while the other contains the 3D anatomical landmarks. The JSON file is used to store the 2D keypoints and the calibration parameters of the virtual cameras. The anthropometric characteristics of the participants are annotated in a single CSV file.

These data are intended to be used in developments that require the transformation of existing human pose solutions in computer vision into biomechanical applications or simulations. This dataset can also be used in other applications related to training neural networks for human motion analysis and studying their influence on anthropometric characteristics.

Specifications Table

**Table:** 

Subject	Computer Science
Specific subject area	The dataset we present combines the results of two different technologies for markerless human motion capture. On the one hand, the human keypoint data are obtained from MediaPipe as a development in the field of human pose estimation from images (computer vision). On the other hand, the anatomical landmark trajectories are obtained from a temporal scanning and processing system that uses dense meshes with anatomical correspondence (computer graphics).
Data format	Raw and processed data
Type of data	JSON files: contain camera projection matrices and human 2D keypoint coordinates detected in the images and their scores. There is one JSON file for each subject and movement. TRC files: contain 33 3D human keypoint coordinates and 53 3D human anatomical landmark coordinates. There are two TRC files for each subject and movement (one corresponding to 3D keypoints and the other to anatomical landmarks).
Data collection	A collection of 3D textured moving meshes was obtained using Move4D, a temporal scanning and processing system. 71 volunteers participated in the scanning sessions where they performed the A-Pose and 7 different movements. For each mesh, 48 images were rendered by virtual cameras. 33 2D keypoints were detected in each image using the MediaPipe Pose Landmarker model. 3D keypoints were triangulated using the cameras’ projection matrices and the 2D keypoints. In addition, 53 anatomical landmarks were obtained for each mesh as the location of fixed vertex indices. Erroneous meshes and 2D keypoints detected with low scores were discarded.
Data source location	Institution: Human Analysis Laboratory (HAL) of Instituto de Biomecánica - IBV City/Town/Region: Valencia Country: Spain. Latitude and longitude: 39.4789124882017, -0.3333209926601934
Data accessibility	Repository name: Mendeley Data Data identification number: 10.17632/493s6f753v.2 Direct URL to data: https://data.mendeley.com/datasets/493s6f753v/2

## 1 Value of the Data

• To the best of the authors' knowledge, this is the first dataset to provide data of the same individuals for pose estimation and motion analysis calculated using different markerless methods (keypoints from images and anatomical landmarks from a temporal scanner). This makes it possible to study their relationship and to train models that compute the joint centers more accurately.
• The dataset contains 2D and 3D data of 567 movement sequences in TRC and JSON files. The data were gathered from a large and anthropometrically diverse group of participants who performed the canonical reference A-Pose and 7 different movements usually employed to evaluate human motion capture systems. The format of the 3D data provided makes them suitable for direct use in simulation platforms and motion capture programs such as OpenSim^Ⓡ^.
• The reconstruction error of the 3D keypoints provided is minimized by the use of 48 virtual cameras.
• The nature of the data allows them to be used for biomechanical purposes such as calculating gait spatio-temporal parameters, joint centers, joint angles, etc. and studying their relationship with the anthropometric characteristics of the population.
• Both keypoints and anatomical landmark trajectories can be used to train predictive neural networks.
• 2D keypoints, scores and camera projection matrices make it possible to study different implementations for pose estimation, such as mono-camera approaches, and to perform 3D reconstructions with different numbers of cameras, selected perspectives or based on 2D keypoint detection scores.

## 2 Background

Human motion capture (MoCap) from images of RGB cameras is a topic of rising interest for animation, computer vision and biomechanics. Ground truth datasets of colour images labelled with anatomical points have emerged during the last years. Many of them provide accurate data obtained from marker-based MoCap systems combined with RGB video data, but these images have the same background and the appearance of the subjects is altered by the markers attached to the body, limiting their usefulness for training generalist neural networks [[1], [2]]. Other datasets provide images that are clean of markers in which body keypoints were manually labelled or have been obtained from markerless MoCap systems [[3], [4]]. These datasets were never designed for biomechanical applications and lacked accuracy as body joints positions do not match from a classical anatomical perspective [5]. Little or no technology has been developed from clean images and accurate anatomical data. In order to bridge the gap between those two types of studies, we have produced a dataset that merges such keypoints obtained from pose detection tools and accurate positions of anatomical landmarks, which could be used to infer the latter from low-cost video systems, as shown in work [6].

## 3 Data Description

**Fig. 1:**
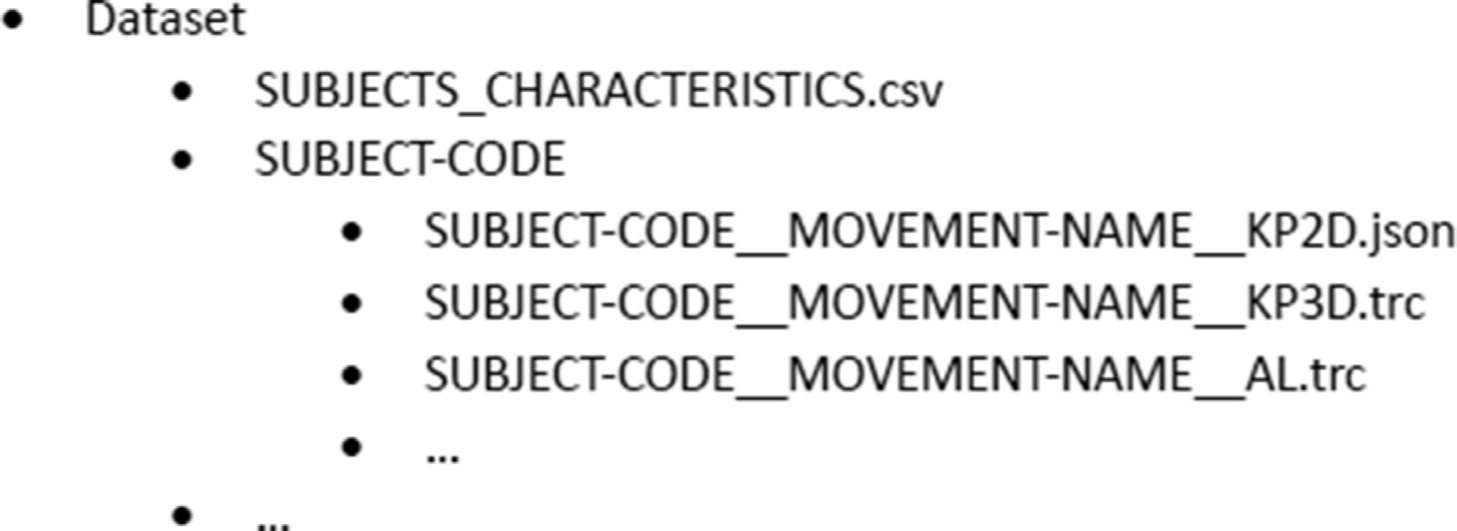
Overview of the dataset folder's structure.

The dataset consists of a CSV file with the anthropometric characteristics of the participants and 71 folders containing the TRC and JSON files of all movement sequences (8 TRC files with anatomical landmarks, 8 TRC files with 3D keypoints and 8 JSON files with 2D keypoints and camera matrices). Fig. 1 shows a general overview of the folder's structure.

### 3.1 CSV file

**Fig. 2:**
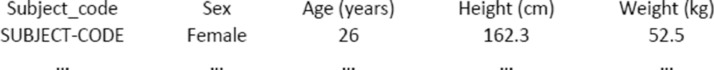
Data in the CSV file of anthropometric characteristics.

The structure of the CSV file is as follows: a header line indicating the data variable and 71 contiguous lines of data corresponding to each subject (Fig. 2).

### 3.2 JSON file

**Fig. 3:**
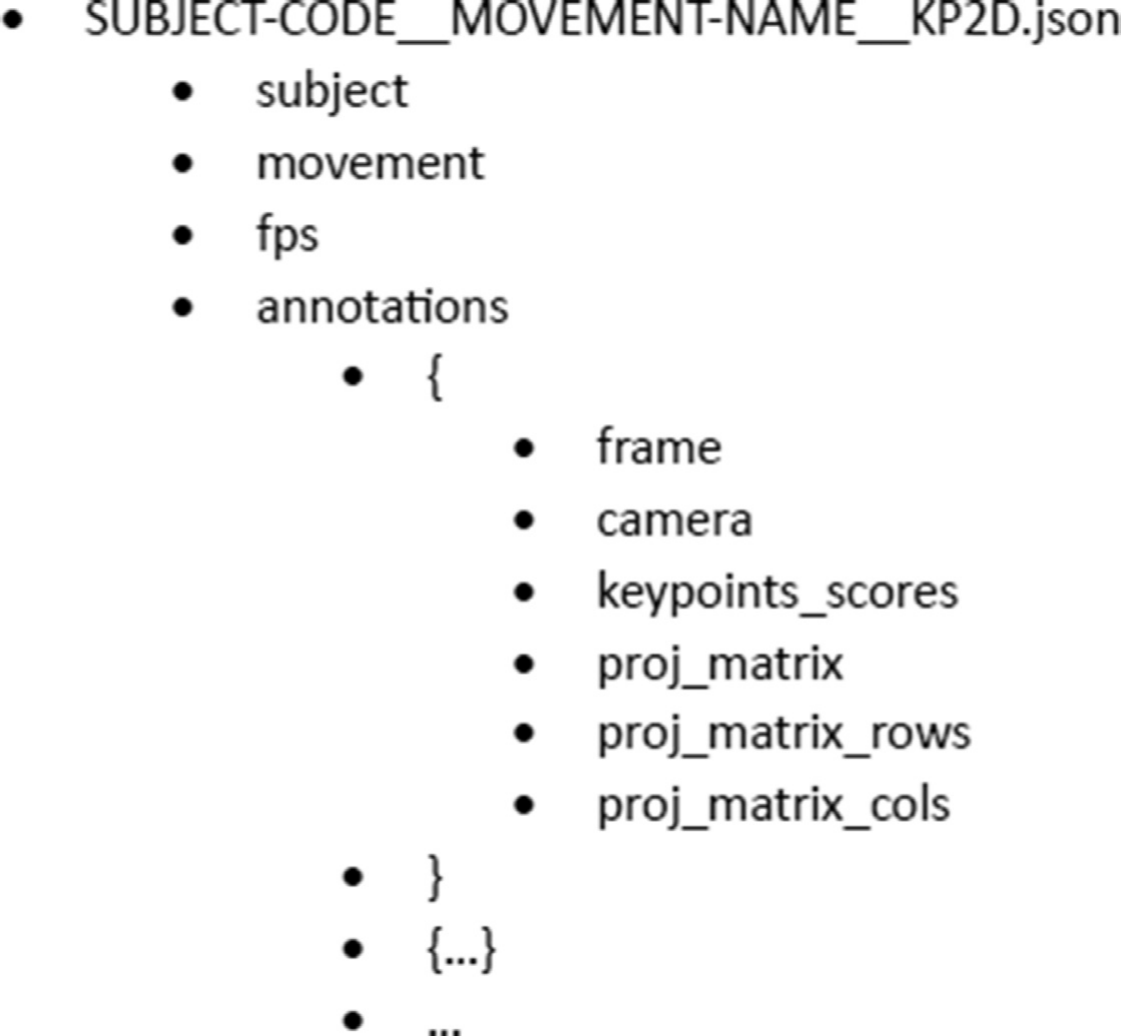
Data structure in JSON file.

Each JSON file (SUBJECT-CODE__MOVEMENT-NAME__KP2D.json) contains general sequence data and a list of annotations. The general data is structured in the fields “subject”, which registers the subject code, “movement”, which contains the movement name, and “fps”, which contains the capture frame rate of this specific movement. Each annotation contains the following fields: “frame”, “camera”, “keypoint_scores”, “proj_matrix”, “proj_matrix_rows” and “proj_matrix_cols”. Fig. 3 shows how the data is organized in the JSON file and Table 1 describes the content of each annotation field.

**Table 1:** Description of fields in the annotation field of the JSON file.

Annotation field	Description
frame	Number of the frame in the movement sequence.
camera	Identification number assigned to the virtual camera.
keypoints_scores	Vector of size 99 (3 × 33) in which data is ordered as (u_1_, v_1_, score_1_, … u_33_, v_33_, score_33_) where (u_i_,v_i_) is the location of the 2D keypoint in pixels and score_i_ is its associated score.
proj_matrix	Vector of size N x M, where N the number of files and M the number of columns.
proj_matrix_rows	Indicates the number of rows in the projection matrix (N = 3).
proj_matrix_cols	Indicates the number of columns in the projection matrix (M = 4).

### 3.3 TRC files

**Fig. 4:**
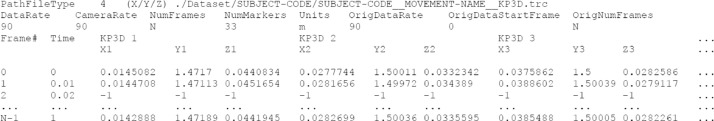
TRC file example.

Two TRC files are provided for each movement sequence. One contains the trajectories of the 3D keypoints (SUBJECT-CODE__MOVEMENT-NAME__KP3D.trc) and the other the trajectories of the 3D anatomical landmarks (SUBJECT-CODE__MOVEMENT-NAME__AL.trc). When the reconstructions failed or there was no mesh for a particular frame, the corresponding line was filled with -1 (see Fig. 4 and the line for frame number 2). The frame sequence starts at 0 and all units are in meters. An example of a TRC file is shown in Fig. 4.

## 4 Experimental Design, Materials and Methods

**Fig. 5:**
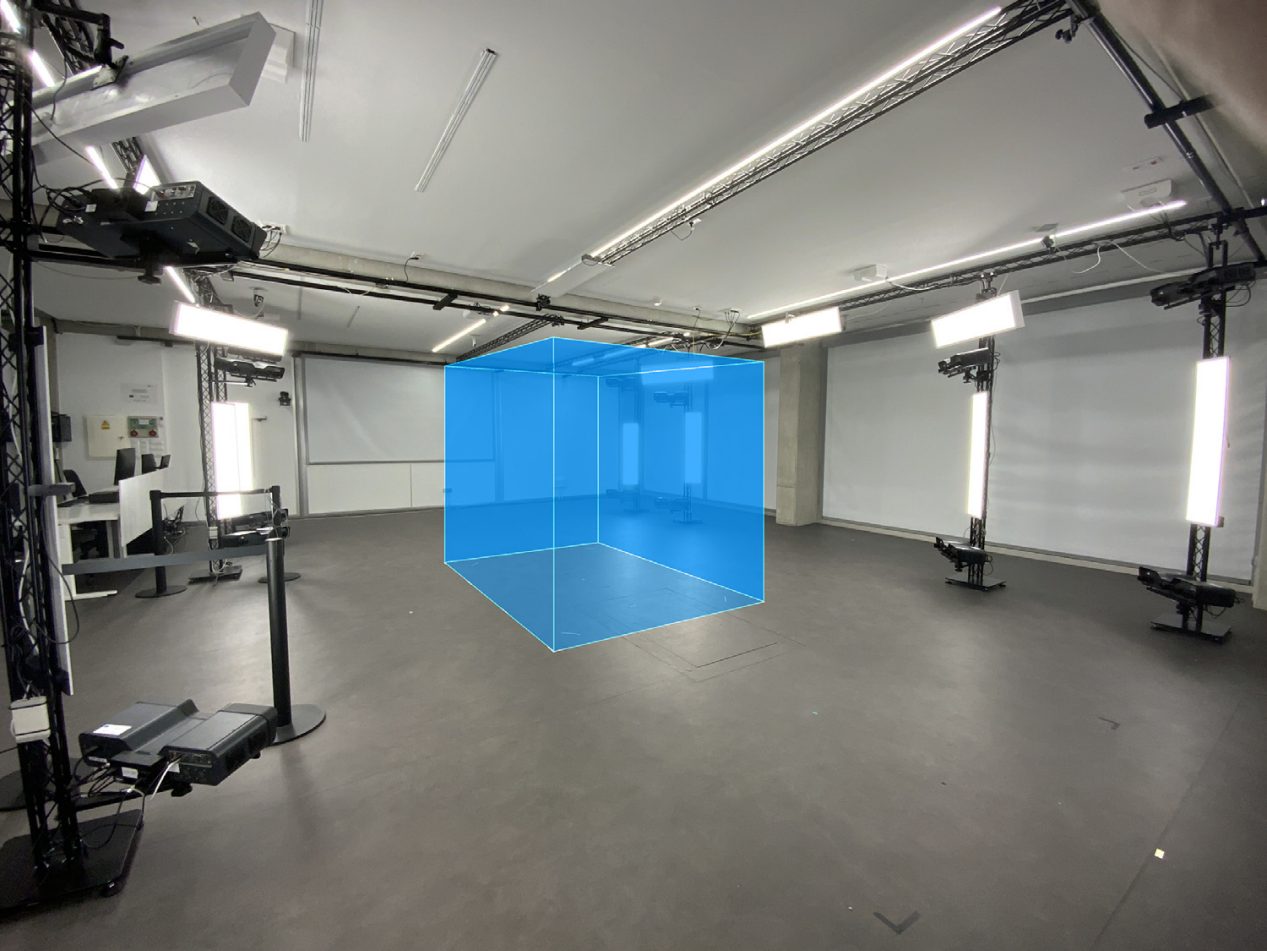
Move4D in the Human Analysis Laboratory (HAL) of the Instituto de Biomecánica - IBV in Valencia. The scanning volume of 3 × 2 × 2.8 meters is shown in blue.

The data were collected in the Human Analysis Laboratory (HAL) of the Instituto de Biomecánica - IBV in Valencia, where the Move4D scanning and processing system is installed, as shown in Fig. 5.

### 4.1 Participants

Seventy-one volunteers (36 women and 35 men) participated in this work. They were selected to provide the maximum variability in terms of the human body. The characteristics of the group of women were limited within the ranges of [20,62] years, [145,180] cm height, [42,120] kg weight and body mass index (BMI) of [17,41] kg/cm2. The characteristics of the group of men were [20,66] years, [163,200] cm height, [51,127] kg weight and BMI of [17,37] kg/cm2. The inclusion criteria for the subjects required that they had no musculoskeletal disorders or other diseases that would prevent them from performing the proposed movements. They all agreed to participate and signed an informed consent form.

### 4.2 Movements

We proposed 7 movements usually performed in biomechanical assessment. In order to prevent problems due to occlusion of body parts, movements that required the support of objects like chairs or stairs were excluded.

Table 2 summarizes the names of the movements, the recording frame rate and the maximum number of frames in the sequence. It also provides a brief description of the movements performed.

**Table 2:** Name, description and characteristics of the movements and poses performed in the dataset.

Movement/pose name	Max. number of frames	Frame rate (fps)	Description
A-POSE	4	90	Canonical pose: upright with legs and arms slightly apart.
J-JACKS	60 (170*)	30 (80*)	The movement corresponds to jumping jacks.
RUNNING	120 (170*)	60 (80*)	Running along the 8 m corridor (5 + 3 m of the scanning system).
GAIT	240 (250*)	60 (50*)	Walking along the 8 m corridor (5 + 3 of the scanning system) at comfortable speed.
JUMP	180 (255*)	60 (80*)	Raise the arms backwards with initial impulse and jump raising the arms as high as possible.
F-JUMP	120	30	Jumping forward.
T-JUMP	90	30	Jumping and turning around at the same time.
SQUATS	120	30	Performing squats while raising the arms in the scapular plane.

⁎ Corresponding values for volunteers TDB_001_F, TDB_002_F and TDB_003_M.

### 4.3 Overview

We first captured people in motion using Move4D [7]. We selected this system for two reasons. First, it has been validated as a good markerless alternative to photogrammetry-based motion capture systems [8] and second, the mesh topology it provides is dense (50k vertices) and has anatomical correspondence with errors comparable to those of manual palpation procedures as explained in [9]. We used the OBJ format to represent the meshes.

**Fig. 6:**
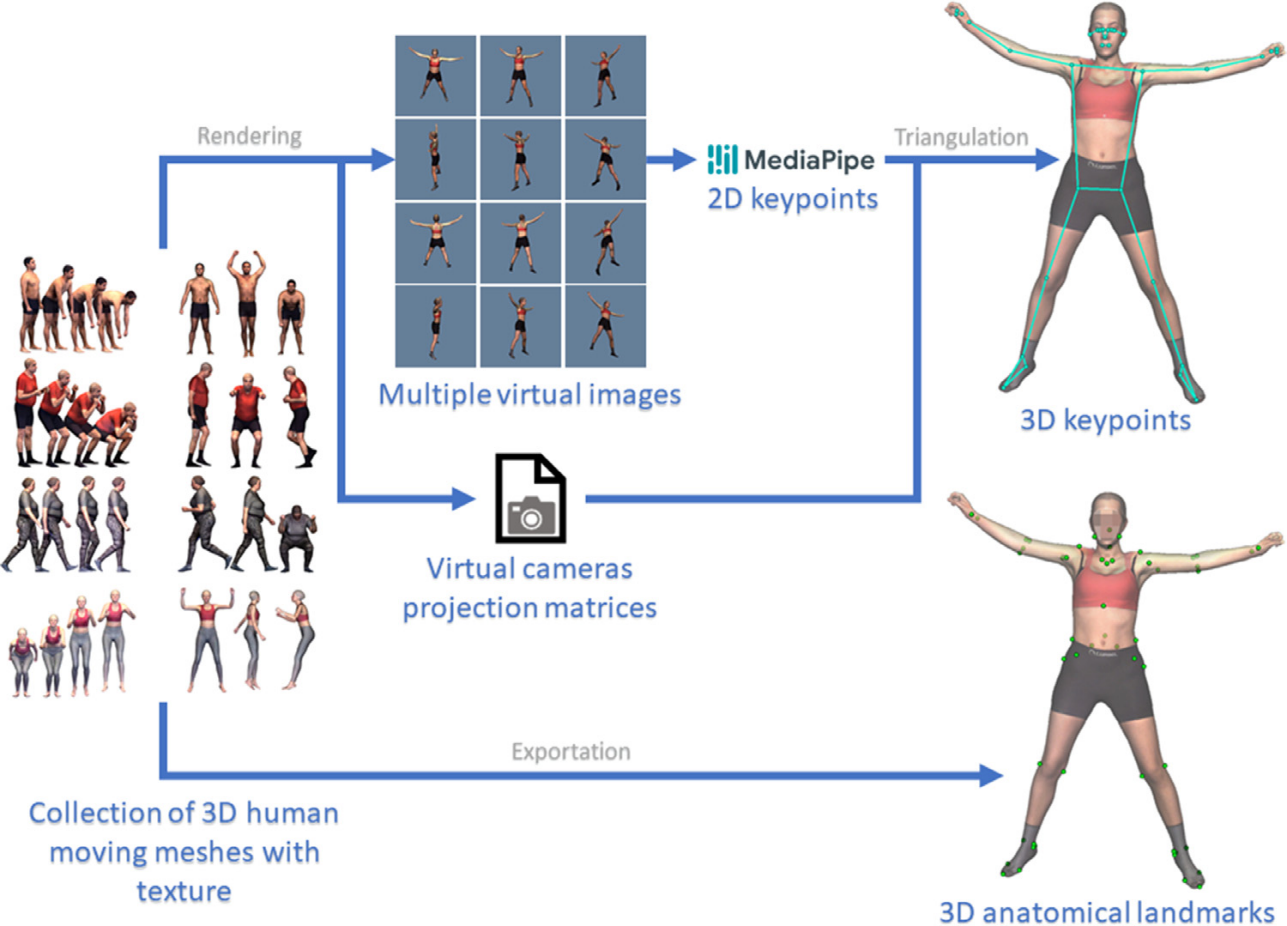
Pipeline followed to obtain this dataset. The upper branch represents the steps followed to obtain the 3D keypoints. The lower branch indicates that AL can be obtain automatically from a homologous mesh.

For each OBJ mesh in the collection, we obtained its 3D anatomical landmarks (AL) location and its 3D keypoints (by triangulating the 2D keypoints from multiple images of the same pose). Fig. 6 shows a scheme of the pipeline followed.

**Fig. 7:**
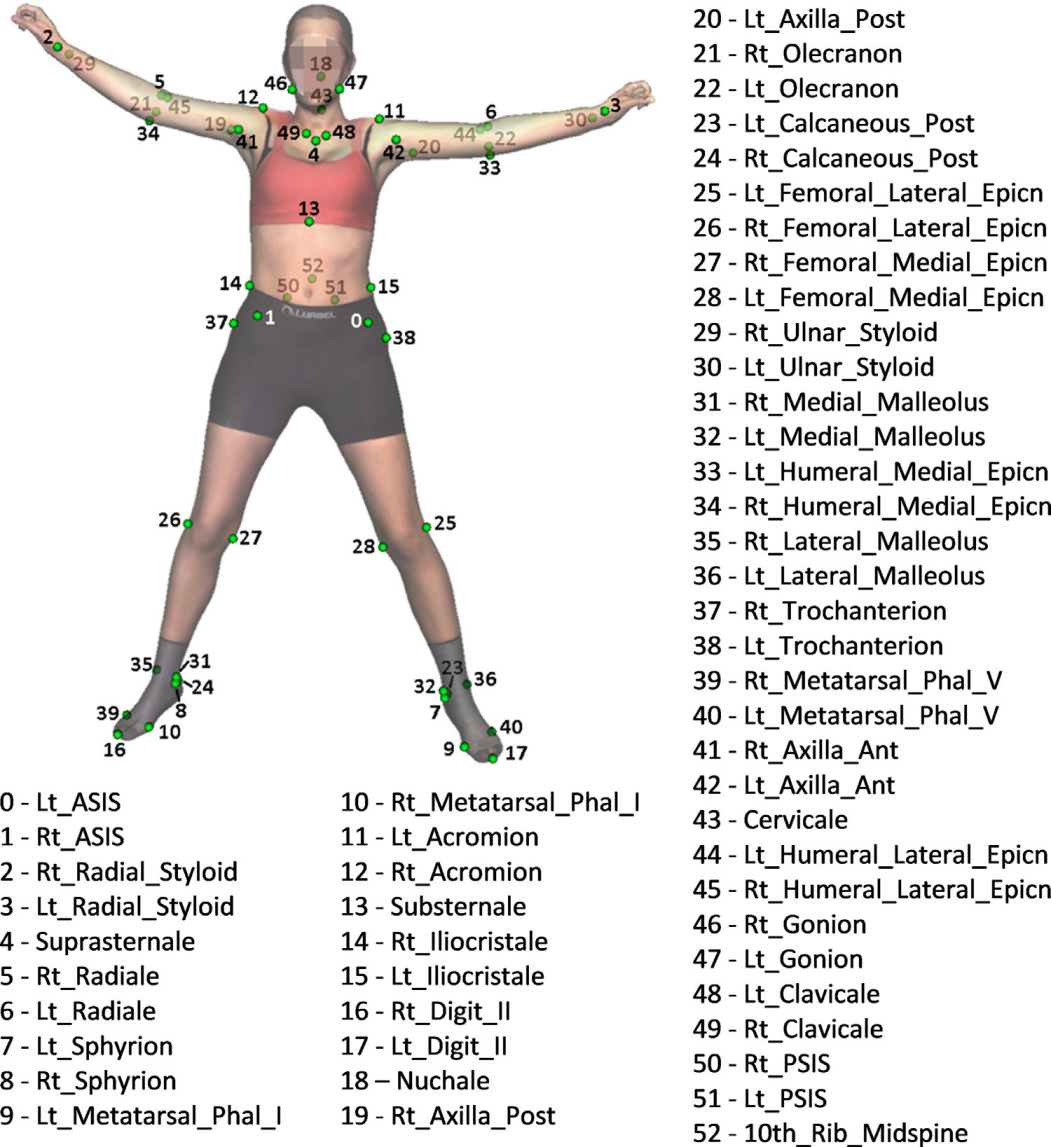
Anatomical landmarks [10]. Left (Lt), right (Rt), phalangeal (Phal), anterior (Ant), posterior (Post), epicondyle (Epicn)

3D AL: Using Move4D, we automatically exported 53 AL coordinates (Fig. 6 lower branch). They corresponded to the coordinates of the fixed vertex indices of the mesh determined in previous work [9]. The indices used were the same for all poses and participants of the same sex (application of the anatomical correspondence property of the mesh). Fig. 7 shows an example of a mesh with all provided AL.

**Fig. 8:**
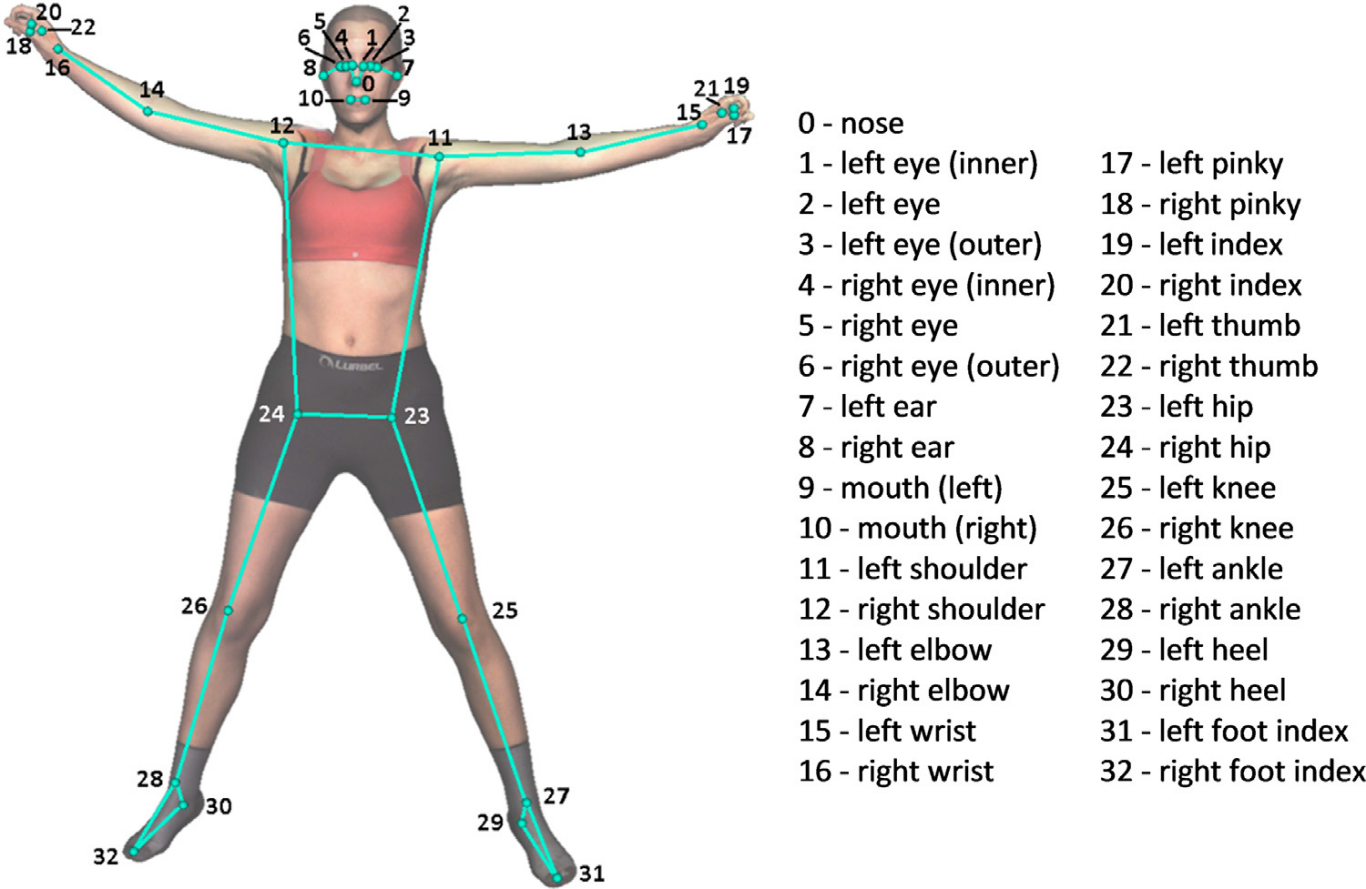
MediaPipe keypoints [12]*.*

3D keypoints: We used each textured mesh to render multiple virtual images and obtained the associated camera projection matrices using the Visualization Toolkit (VTK). We later detected the 33 corresponding 2D keypoints for all the virtual images, with MediaPipe. Finally, we triangulated the 2D keypoints and obtained their 3D keypoints [11]. Fig. 8 shows an example of a mesh with all 3D keypoints provided.

### 4.4 Materials

Move4D: This 4D scanning and processing system [7] captures temporal sequences of 3D body scans and provides textured body meshes. It is a modular photogrammetry-based system consisting of 16 modules covering a scanning area of 3 × 2 × 2.8 m (length x width x height). The processing software converts the raw 3D data into a noise- and artefact-free watertight dense mesh (50K vertices) with texture (4096 × 4096 pixels) that has vertex-to-vertex (anatomical) correspondence. This system was used to scan, process and export the OBJ and TRC files of 3D anatomical landmarks.

The Visualization Toolkit (VTK): It is a software system extensively used for 3D computer graphics, that makes it possible to render volumes and process images [13]. We used VTK to automatically locate the virtual cameras and render multiple virtual images for each textured body mesh (OBJ).

MediaPipe Pose Landmarker Model: This model lets the user find people and body positions in an image or video. It uses machine learning models and provides image coordinates and 3D world coordinates [12]. We used it to detect the 2D pose keypoints in the virtual images (image coordinates).

### 4.5 Methods

#### 4.5.1 Procedure followed to obtain 3D textured human meshes

We scanned each participant during a session. Previously, we calibrated the Move4D system according to the manufacturer's instructions. During the session, the protocol set out below was followed:

1. The participants put on tight clothing, removed their footwear and covered their head with a cap. Their hair was tied back if necessary.
2. We recorded the age of each participant and measured their height and weight using a calibrated stadiometer and scale.
3. We scanned the participant in the reference pose, the canonical A-Pose.
4. We scanned the participant performing 7 different movements.
5. Finally, we launched the automatic processing integrated in Move4D.
6. All captured and processed 3D data were reviewed by human experts to detect and remove possible errors in the capture or processing of the scans. This activity was a visual inspection for checking that pose and shape of the homologous mesh fitted the point cloud. An example is shown in Fig. 9.

**Fig. 9:**
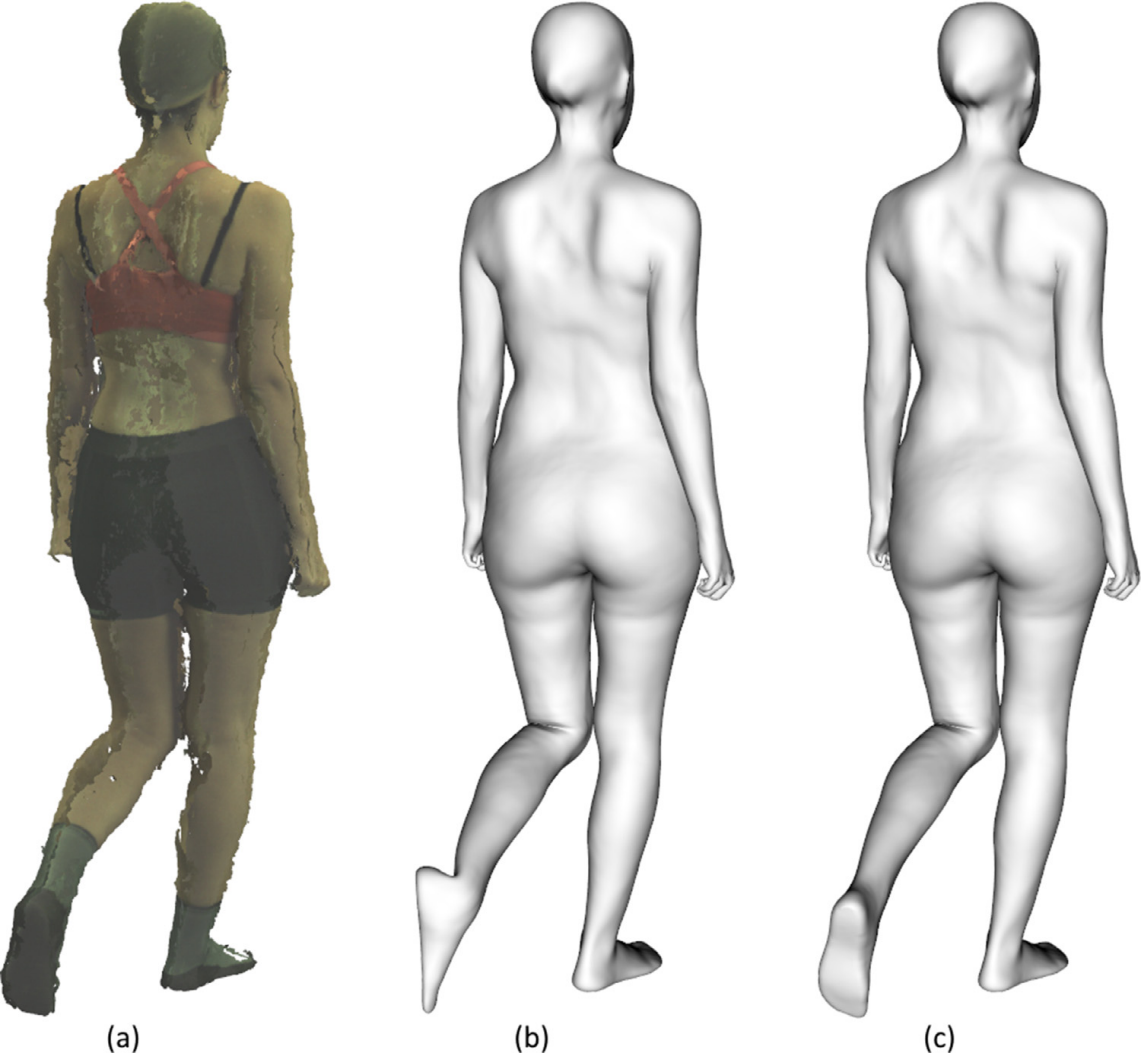
Example of good and bad processing result: (a) raw point cloud (b) bad processing of left toe (c) good processing.
7. We then exported these data in a standard and compatible OBJ format with a PNG texture map.

The jump scan of participant TDB_013_M was corrupted and discarded. Some frames in gait and running scans were empty or lacked certain body parts because participants had been instructed to start the movement outside the scan volume and to then enter and cross it afterwards. Those frames and the frames removed due to processing failure (a total of 15,917) are not included in the 51,051 poses referred to above. We finally obtained 567 movement sequences in total, including the A-Pose.

Volunteers TDB_001_F, TDB_002_F and TDB_003_M were scanned with different parameter settings (number of frames and frame rate). After scanning and checking the preliminary results of these 3 volunteers, we decided to change the parameter values of some movements to more suitable ones.

#### 4.5.2 Procedure followed to obtain multiple virtual images and projection matrices

For each textured mesh, 48 PNG images corresponding to 48 viewpoints were rendered using virtual cameras with VTK. The virtual cameras were configured to point at the center of the 3D bounding box of the mesh and were arranged in 3 rings of 16 cameras each at different heights.

The image size was designed to have the same height and width, and its magnitude depended on the mesh. In the majority of cases, their size was around 450 × 450 pixels.

The virtual cameras projection matrices were noted in the correspondent JSON file.

#### 4.5.3 Procedure followed to obtain the 2D keypoints

The 2D keypoints were detected using MediaPipe for each image of the mesh. The MediaPipe model selected to achieve these results was the Pose Landmarker model. The 2D keypoints as well as their associated scores were noted in the corresponding JSON.

For each participant and movement sequence, we provide a JSON file with the virtual camera projection matrices and the 2D keypoints with their scores.

#### 4.5.4 Procedure followed to obtain the 3D keypoints

The corresponding 3D keypoint coordinates were obtained by linear triangulation [11] from the 2D keypoints of all the images of each pose. The reconstruction of each keypoint was done with a previously determined optimal combination of virtual cameras.

To do this, we first selected the keypoints detected with higher scores and reviewed the MediaPipe scores of all 2D keypoints, ignoring all those with scores below 0.5. We also discarded any poses where the average score of all 2D keypoints in all images did not exceed 0.6.

We then calculated the epipolar errors for each set of cameras and 2D keypoint, discarding those with errors above 3 pixels.

Once we had determined the set of cameras with low epipolar errors for each 2D keypoint, we applied a linear triangulation to obtain the 3D keypoints, which were registered in the corresponding TRC file.

For each participant and movement sequence, we provide a TRC file with the 3D keypoints.

#### 4.5.5 Procedure followed to obtain the 3D anatomical landmarks

We exported the 3D coordinates of 53 AL as the coordinates of fixed mesh vertices that better represented them with Move4D. The details of the procedure followed to determine these fixed vertices is explained in [9].

For each participant and movement sequence, we provide a TRC file with the 3D AL.

## Limitations

This work was carried out in a controlled environment in which the participants were required to wear tight clothing and caps. Also, certain gestures could not be recorded as they require the use of objects (a chair to sit on, steps to climb, etc.). The introduction of objects into the scanning system would have masked the body surfaces resulting in poor and incomplete raw scans.

## Ethics Statement

This work was approved by the Ethics Committee of the Universitat Politècnica de València (P5_05_03_20) and conducted in accordance with the Declaration of Helsinki.

## CRediT authorship contribution statement

**Ana Virginia Ruescas-Nicolau:** Conceptualization, Software, Validation, Investigation, Resources, Data curation, Writing – original draft. **Enrique José Medina-Ripoll:** Conceptualization, Methodology, Software, Validation, Investigation, Data curation, Supervision. **Eduardo Parrilla Bernabé:** Conceptualization, Methodology, Writing – review & editing, Supervision. **Helios de Rosario Martínez:** Conceptualization, Writing – review & editing, Supervision.

## Data Availability

Human tracking dataset of 3D anatomical landmarks and pose keypoints (Original data) (Mendeley Data).
